# Repression of human activation induced cytidine deaminase by miR-93 and miR-155

**DOI:** 10.1186/1471-2407-11-347

**Published:** 2011-08-10

**Authors:** Glen M Borchert, Nathaniel W Holton, Erik D Larson

**Affiliations:** 1 School of Biological Sciences, Illinois State University, Normal, IL 61790-4120, USA

**Keywords:** AICDA, AID, CSR, hypermutation, microRNA, miR-93, miR-155, SHM, UTR, 3'UTR

## Abstract

**Background:**

Activation Induced cytidine Deaminase (AID) targets the immunoglobulin genes of activated B cells, where it converts cytidine to uracil to induce mutagenesis and recombination. While essential for immunoglobulin gene diversification, *AID *misregulation can result in genomic instability and oncogenic transformation. This is classically illustrated in Burkitt's lymphoma, which is characterized by AID-induced mutation and reciprocal translocation of the *c-MYC *oncogene with the IgH loci. Originally thought to be B cell-specific, *AID *now appears to be misexpressed in several epithelial cancers, raising the specter that AID may also participate in non-B cell carcinogenesis.

**Methods:**

The mutagenic potential of AID argues for the existence of cellular regulators capable of repressing inappropriate *AID *expression. MicroRNAs (miRs) have this capacity, and we have examined the publically available human *AID *EST dataset for miR complementarities to the human *AID *3'UTR. In this work, we have evaluated the capacity of two candidate miRs to repress human AID expression in MCF-7 breast carcinoma cells.

**Results:**

We have discovered moderate miR-155 and pronounced miR-93 complementary target sites encoded within the human *AID *mRNA. Luciferase reporter assays indicate that both miR-93 and miR-155 can interact with the 3'UTR of *AID *to block expression. In addition, over-expression of either miR in MCF-7 cells reduces endogenous AID protein, but not mRNA, levels. Similarly indicative of *AID *translational regulation, depletion of either miR in MCF-7 cells increases AID protein levels without concurrent increases in *AID *mRNA.

**Conclusions:**

Together, our findings demonstrate that miR-93 and miR-155 constitutively suppress *AID *translation in MCF-7 cells, suggesting widespread roles for these miRs in preventing genome cytidine deaminations, mutagenesis, and oncogenic transformation. In addition, our characterization of an obscured miR-93 target site located within the *AID *3'UTR supports the recent suggestion that many miR regulations have been overlooked due to the prevalence of truncated 3'UTR annotations.

## Background

In antigen activated B cells the Activation Induced cytidine Deaminase (AID) protein is required for initiating mutagenesis and recombination of the immunoglobulin (Ig) genes to promote immunity. AID is a cytidine deaminase that converts single-stranded genomic cytidine into uracil, and its activity is pronounced at the Ig variable (V) and switch (S) regions [1-5]. While spontaneously generated uracil in the genome is faithfully corrected as a part of normal DNA repair [6], AID-induced uracils at the Ig loci are mutagenically resolved by multiple DNA repair factors [4,7]. Although AID targets the Ig loci in activated B cells, activity at other genomic sites has the potential to create oncogenic DNA damage. Indeed, transcribed genes across the genome have now been found to undergo AID deamination (reviewed by [8,9]), and recently, deep sequencing of AID-ChIPed template revealed broad AID-associations with expressed loci [10]. In addition, AID-dependent DNA breaks were recently identified within multiple types of repetitive elements [11] suggesting a broad ability for AID to target genomic regions harboring single-stranded character.

*AID *misexpression results in DNA damage that promotes cancer [11-16]. In lymphoid cells improper *AID *expression has been connected with mutations at *c-MYC*, *PIM1*, *RhoH *and *PAX5 *oncogenes, promoting diffuse large B cell lymphoma [17]. Similarly, Burkitt's lymphoma is characterized by AID-induced mutation and a reciprocal translocation between the *c-MYC *proto-oncogene and the IgH loci [9,18-20]. While *AID *expression was initially thought to be B cell-specific, recent evidence indicates AID may promote the development of various non-lymphoid oncologies. In gastric cancer, the upregulation of *AID *leads to point mutations and copy number alterations of CDKN2A and CDKN2B tumor suppressor genes [21]. In the same manner, *AID *misexpression in human colonic cells increases the mutation rate of *TP53 *by ~10-fold [22]. In addition, *AID *is expressed at various levels in ~1/3 of primary lung cancers [23] and in numerous breast cancer cell lines [24] (where *AID *misexpression may be due in part to observations that estrogen is capable of inducing *AID *expression > 20 fold [25]). Since errant AID activity can introduce significant genomic damage, the maintenance of genome stability outside of the activated B cell environment likely depends upon multiple molecular AID restraints.

Recent evidence suggests that one level of *AID *regulation comes from microRNAs (miRs). These short, ~20 nt, noncoding RNAs can regulate networks of genes and function by repressing translation or directing mRNA destruction through partial sequence complementarity to 3' untranslated regions (3'UTRs) of mRNAs [26,27]. MiRs are key regulators of cellular differentiation, proliferation and apoptosis, and aberrant miR expression has been associated with a myriad of human diseases, including cancer (reviewed by [28]). A subset of miRs typically misexpressed in malignancy (oncomiRs) can function as oncogenes or tumor suppressors with impacts on cellular transformation and metastasis. One such oncomiR, miR-155, represses murine *AID*, and the disruption of the miR-155 recognition site or miR-155 itself results in increased AID-induced *c-MYC *translocation [29] and *BCL6 *mutagenesis [30]. This may provide one explanation for why disruption of miR-155 is associated with Burkitt's lymphoma [31] and suggests the existence of parallel miR repressions in humans.

In order to better define the regulators of human *AID *in cells other than antigen-activated B cells, we examined the 3'UTR of human *AID *for miR complementarities. Consistent with the role of miR-155 in regulating *AID *in mice [29,30], we find a moderate miR-155 site in human *AID*. Surprisingly, we also find a previously uncharacterized, yet exceptional, complementarity to miR-93 in the *AID *3'UTR. We show both of these miRs interact with the *AID *3'UTR in the cell to regulate its translation, and that loss of either miR results in increased AID protein levels. Based on the involvement of AID in generating oncogenic genome mutations, our results suggest that miR-93 and miR-155 act as dual genome sentries to prevent errant translation of *AID *mRNA.

## Methods

### Reagents and cell lines

Oligonucleotide sequences are detailed in Additional File 1, Table S1. Human embryonic kidney (HEK293) cell line was obtained from GenLantis (San Diego, CA), Burkitt's lymphoma (Ramos) and breast cancer (MCF-7) cells were both purchased from the American Type Culture Collection, (ATCC, Manassas, VA). HEK293 and MCF-7 cells were cultured in MEM (Mediatech, Herndon, VA) supplemented with 10% fetal bovine serum (Hyclone, Logan, UT). Ramos cells were cultured in RPMI (Mediatech) supplemented with 10% fetal bovine serum (Hyclone, Logan, UT). All tissue culture media were supplemented with 25 mg/ml streptomycin and 25 I.U. penicillin (Mediatech). Cells were cultured in a humidified atmosphere with 5% CO2 at 37°C. For luciferase assays, HEK293 s were cultured in MEM (10% FBS and 1% PS) in 12-well plates. At 90% confluency, cells were transfected following the Lipofectamine 2000 (Invitrogen, Carlsbad, CA) protocol. At 35 h, existing media was replaced with 1 ml fresh media. For miR expression assays, MCF-7 s were cultured in MEM (10% FBS and 1% PS) in 6-well plates. At 90% confluency, cells were transfected following the Lipofectamine 2000 (Invitrogen) protocol and harvested after 48 h. For miR sponge assays, MCF-7 s were cultured in MEM (10% FBS and 1% PS) in 6-well plates. At 70% confluency, cells were transfected following the Lipofectamine 2000 (Invitrogen) protocol. At 48 h, existing media was replaced with 2 ml fresh media and cell transfections were repeated as initially performed. Cells were then harvested after an additional 48 h culture.

### Luciferase assays

At 36 h post transfection, cells were scraped from well bottoms and transferred to 1.5 ml Eppendorf tubes. Eppendorfs were centrifuged at 2000 RCF for 3 min, followed by supernatant aspiration and cell resuspension in 300 μl of PBS. Cells were lysed by freeze thaws and debris removed by centrifuging at 3000 RCF for 3 min. 50 μl of supernatant was transferred to a 96-well MicroLite plate (MTX Lab Systems, Vienna, VA) then firefly and *Renilla *luciferase activities measured using the Dual-glo Luciferase^® ^Reporter System (Promega, Madison, WI) and a 96-well plate luminometer (Dynex, Worthing, West Sussex, UK). RLUs were calculated as the quotient of *Renilla*/firefly RLU and normalized to mock.

### Western blotting

MCF-7 cells (at ~1 × 10^6 ^cells/ml) were pelleted by centrifugation, existing media removed, and cells resuspended in SDS lysis buffer containing protease inhibitors and transferred to 1.5 ml Eppendorf tubes. Proteins were electrophoresed through a 4-12% SDS-polyacrylamide gradient gel (Invitrogen) and transferred to immobilon-P PVDF membranes (Millipore, Temecula, CA). Membranes were blocked for 1 hour in 5% (w/v) nonfat milk in phosphate-buffered saline containing 0.05% Tween 20, washed, and incubated with primary antibody overnight at 4°C using the following dilution: anti-AID (Santa Cruz Biotechnology, Santa Cruz, CA, sc-25620) - 1:500 and anti-PCNA (Santa Cruz) - 1:10000. Membranes were washed and incubated with secondary Abs: HRP conjugated goat anti-mouse and goat anti-rabbit (Invitrogen) at 1:10000 dilution. Immunoreactive bands were visualized with ECL Plus (GE, Piscataway, NJ) and signals were detected by using the Storm 840 PhosphorImager and IMAGEQUANT software (GE).

### Vector construction

Unless otherwise indicated, PCR amplifications were performed in 40 μl reactions at standard concentrations (1.5 mM MgCl_2_, 0.2 mM dNTP, 1× Biolase PCR buffer, 0.5 U Taq (Bioline USA, Inc., Randolph, MA), 0.5 uM each primer) and using standard cycling parameters (94°C - 3 min, (94°C - 30 s, 55°C - 30 s, 72°C - 60 s) × 30 cycles, 72°C - 3 min) then cloned into Topo PCR 2.1 (Invitrogen). RT-PCRs were performed at 65°C using MonsterScript Reverse Transcriptase (#MSTA5110, Epicentre, Madison, WI) and gene specific or random nonamer primers. Resultant amplicons were cloned into Topo PCR 2.1 and sequenced. Antisense reporter, Ctl 3'LR, was constructed by oligonucleotide primer extension (25 cycles with 10 s extensions) with primers containing 5' Xho-I and 3' Spe-I restriction enzyme sites immediately flanking sequences perfectly complementary to mature siLacZ. Antisense reporter, AID 3'LR, was constructed by standard PCR with primers containing 5' Xho-I and 3' Spe-I restriction enzyme sites. Following digestion, amplicons were ligated into the *Renilla *luciferase 3'UTR of psiCheck2 (Promega) vector linearized with Xho-I and Spe-I. The presence of an independently transcribed firefly luciferase in these reporters allowed normalization for transfection efficiency. Sponge expression constructs were generated by concatamerizing PCR using the primers indicated in Additional File 1, Table S1. Resulting amplicons were separated on a 1% agarose gel and a band excised from the appropriate lane at ~400 bp. Gel extractions were cloned into Topo PCR 2.1 and sequenced. Concatamers were next excised from Topo PCR 2.1 and cloned into the pEGFP expression vector (Clontech, Mountain View, CA) using BamHI and Not I restriction sites.

## Results

### The human *AID *3'UTR contains sequences complementary to miR-93 and miR-155

**Figure 1:**
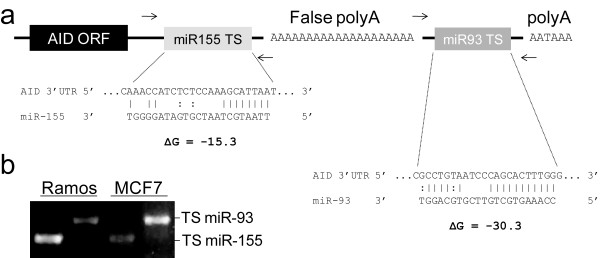
**The *AID *3'UTR**. (**A**) Cartoon of *AID *3'UTR miR target sites (TS) and internal (False) polyA (not to scale) (DNA sequence shown). MicroRNA alignments are shown with corresponding binding affinities (kcal/mol). Vertical lines indicate complementary base pairing. Dotted vertical lines indicate G:U base pairing. PolyA, AATAAA eukaryotic polyadenylation signal. Arrows indicate RT-PCR primer sets for confirmation of expression. (**B**) RT-PCR to test expression of the *AID *3'UTR miR-155 and miR-93 target site (TS) sequences in Ramos and MCF-7 cells.

In light of the evidence supporting *AID *regulation by miR-155 in mouse showing miR-155 regulates AID translation and c-myc translocation [29,30], we asked if human *AID *is regulated by similar microRNA interactions. Computational analysis of the human *AID *3'UTR revealed a human miR-155 target site, consistent with studies in mice [29,30]. To our surprise, we also found pronounced complementarity between the *AID *3'UTR and miR-93 (Figure 1A). This miR-93 target site is markedly more extensive than the majority of characterized miR::mRNA interactions containing only a single, centrally located 4 bp mismatch. Strikingly, we find the predicted binding affinity between miR-93 and the *AID *3'UTR (-30.3 kcal/mole) to be nearly double that of miR-155 (-15.3 kcal/mole) (Figure 1A) suggesting a strong post-transcriptional regulatory relationship. Further analysis of the human *AID *3'UTR revealed an internal 20 nt stretch of adenosines (sufficient for cDNA poly-T priming) separating the miR-155 and miR-93 target sites (Figure 1A). This false polyA is located ~1,000 nt 3' of the *AID *stop codon and does not correspond to the full length cDNA polyadenylation as: (1) it is not preceded by the canonical 5' AAUAAA poly adenylation signal or other recognizable alternative poly adenylation signal, and (2) the miR-93 target site is expressed in both Ramos and MCF-7 cells (Figure 1B). Importantly, examination of publically available EST datasets confirms this internal polyA stretch has repeatedly served as an oligo-dT priming site for EST reverse transcriptase leading to the improper annotation of a truncated *AID *cDNA (for example see NCBI accessions GI:33871601 and GI:50496022). We conclude that the human *AID *3'UTR contains miR-155 and miR-93 recognition sites, which are separated by a 20 nucleotide adenosine repeat upstream from the true polyA sequence.

### MiR-93 and miR-155 interact with the *AID *3'UTR to inhibit its expression

**Figure 2:**
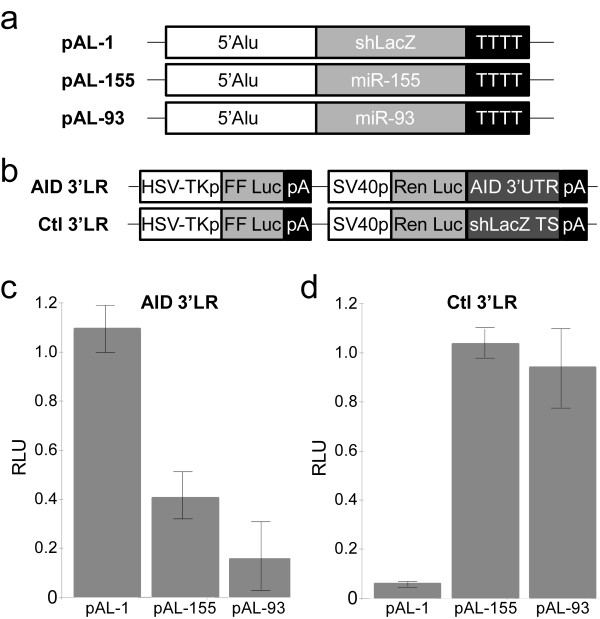
**The *AID *3'UTR is recognized by miRs -93 and -155**. (**A**) Diagrams of miR and siRNA expression vectors. 5'Alu corresponds to a genomic fragment containing the miR-517a upstream Alu promoter [32]. In pAL-1, shLacZ replaces the miR-517a hairpin. In pALs-155 and -93, the pre-miR-155 and -93 hairpins replace the miR-517a hairpin respectively. TTTT, RNA polymerase III termination signal. (**B**) Diagrams of luciferase reporters. The *AID *3'UTR luciferase reporter (AID 3'LR) and control 3'UTR luciferase reporter (Ctl 3'LR) were generated respectively by placing an *AID *3'UTR sequence containing both the miR-155 and miR-93 target sites and a specific region of LacZ previously shown to be targeted by the pAL-1 shLacZ hairpin in a multiple cloning site in the *Renilla *luciferase 3'UTR [32]. HSV-TKp, herpes simplex virus thymidine kinase promoter; SV40p, simian virus 40 promoter; FF Luc, firefly luciferase; Ren Luc, *Renilla *luciferase; pA, poly(A). (**C**) AID 3'LR is specifically repressed by both miR-93 and miR-155 in 293 transient transfections. Luciferase assays (n = 4) of HEK293 lysates after transfection of AID 3'LR and either pAL-1, pAL-155 or pAL-93. Transfections were normalized to AID 3'LR alone. RLU, relative light units. (**D**) Ctl 3'LR is specifically repressed by the ctl (pAL-1) in 293 transient transfections. Cotransfections done as in (**C)**, except Ctl 3'LR replaced *AID *3'LR.

The identification of miR-93 and miR-155 binding sites in the 3'UTR of human *AID *strongly suggests post-transcriptional regulatory interactions. Therefore, we next asked if miR-93 and miR-155 were capable of regulating the *AID *3'UTR using standard luciferase reporter assays. Three expression constructs incorporating an endogenous human microRNA Alu promoter were created by cloning the endogenous miR-517a genomic locus and then replacing the miR-517a hairpin with the miR-93, miR-155 and shLacZ hairpins (pAL-93, pAL-155, and pAL-1 respectively)(Figure 2A), as described [32]. We also generated 3'UTR reporter constructs to test *AID*::miR regulatory interactions. These reporters (AID 3'LR and Ctl 3'LR) produce *Renilla *luciferase transcripts tailed with the *AID *3'UTR and shLacZ target sequence respectively (Figure 2B). Co-transfection of human embryonic kidney (HEK293) cells with pAL-93 and AID 3'LR constructs resulted in ~80% knockdown of *Renilla *luciferase intensity, whereas co-transfection of pAL-155 and AID 3'LR constructs resulted in ~60% knockdown of *Renilla *luciferase intensity at a 4:1 transfection ratio (Figure 2C). Similarly and as expected, co-transfection of pAL-1 and Ctl 3'LR resulted in a reduction of *Renilla *luciferase intensity with ~95% knockdown (Figure 2D). In contrast and as expected, neither the co-transfection of pAL-1 with AID 3'LR nor co-transfection of either miR construct with Ctl 3'LR resulted in silencing (Figure 2C-D). To further ensure the repressions of AID 3'LR by pAL-93 and pAL-155 were due to specific interactions between miRs -93 and -155 and their proposed target sites in the AID 3'UTR, we constructed two additional luciferase reporters whose 3'UTRs consisted of: (1) the two target sites with intervening sequence removed and (2) scrambled versions of the two target sites with intervening sequence removed. Again, we found co-transfection of human embryonic kidney (HEK293) cells with pAL expression constructs and a luciferase reporter containing intact miR target sites resulted in significant repression of Renilla luciferase in contrast to control transfections of scrambled miR target site reporters, which were not repressed (Additional File 2, Figure S1). Therefore, miR-93 and miR-155 facilitate molecular interactions with the *AID *3'UTR sufficient to repress translation.

### MiR-93 and miR-155 can repress endogenous *AID*

**Figure 3:**
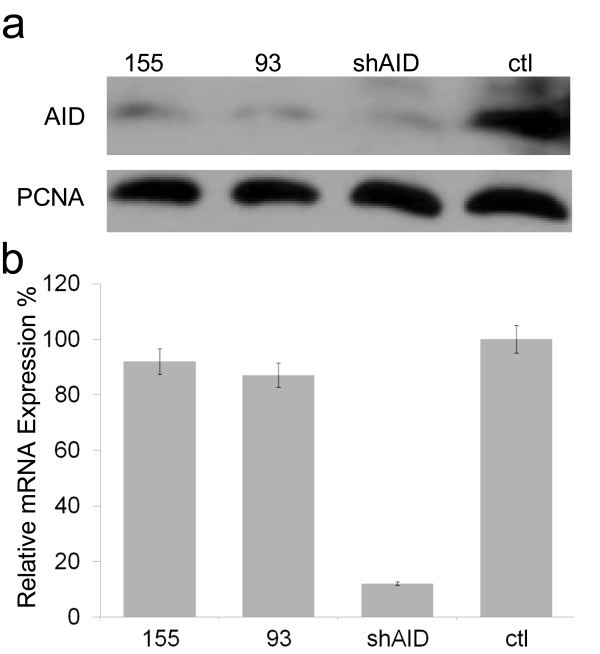
**MiR-93 and miR-155 can repress endogenous *AID***. (**A**) Representative western demonstrating that transient transfection of pAL-93, pAL-155 and shAID expression constructs repress endogenous AID protein levels in MCF-7 cells. ShAID produces an AID-targeting siRNA from a pAL-driven hairpin expression vector as in **2A**. Ctl, pAL-1 transfected; PCNA, Proliferating Cell Nuclear Antigen load control. (**B**) Relative *AID *mRNA levels corresponding to replicate transfections performed as in **A **(n = 3) as determined by quantitative PCR.

Interactions between miR-93, miR-155 and the *AID *3'UTR within the context of our reporter assays (Figure 2C-D) strongly support the involvement of both miRs in *AID *translational regulation. If so, we anticipated that if miR-93 and/or miR-155 were present in *AID *expressing cells they could suppress endogenous AID protein levels. We therefore asked if miR-93 and miR-155 are present in two cell lines known to express *AID*, Ramos and MCF-7. Ramos is a B cell line that undergoes constitutive AID-induced somatic hypermutation [33], and MCF-7 is a breast carcinoma that misexpresses *AID *[24]. Using standard RT-PCR analysis, we found miR -93 and -155 transcripts are expressed in both cell lines (Additional File 3, Figure S2), consistent with the ubiquitous expression documented for both of these miRs [34]. In order to determine if endogenous *AID *can be repressed by increased miR-93 and/or miR-155 expression, we introduced pAL miR over-expression plasmids (Figure 2A) into MCF-7 cells [32]. Importantly, overexpression of miR-93, miR-155, or an *AID*-targeting siRNA (shAID) in MCF-7 cells each reduced AID protein levels between 70 and 90%, whereas overexpression of the pAL-1 control did not significantly alter *AID *expression (Figure 3A). To ensure results were the consequence of miR regulation, we confirmed the miR-93 repression of *AID *was dose-dependent (Additional File 4, Figure S3) and isolated total RNA from each of our transfections to monitor *AID *mRNA levels by quantitative PCR. Strikingly, while shAID transfection averaged a 92% reduction of *AID *mRNA levels, neither pAL-93 nor pAL-155 transfection resulted in significant reduction of *AID *mRNA (Figure 3B) confirming that the observed reductions in AID protein levels following their transfections are mediated through translational repression. To summarize, our results demonstrate that the decreases in AID protein upon shAID transfection were due to message degradation, whereas pAL-93 and pAL-155 overexpression resulted in miR-mediated translational repression.

### Endogenous miR-93 and miR-155 restrain AID protein translation

**Figure 4:**
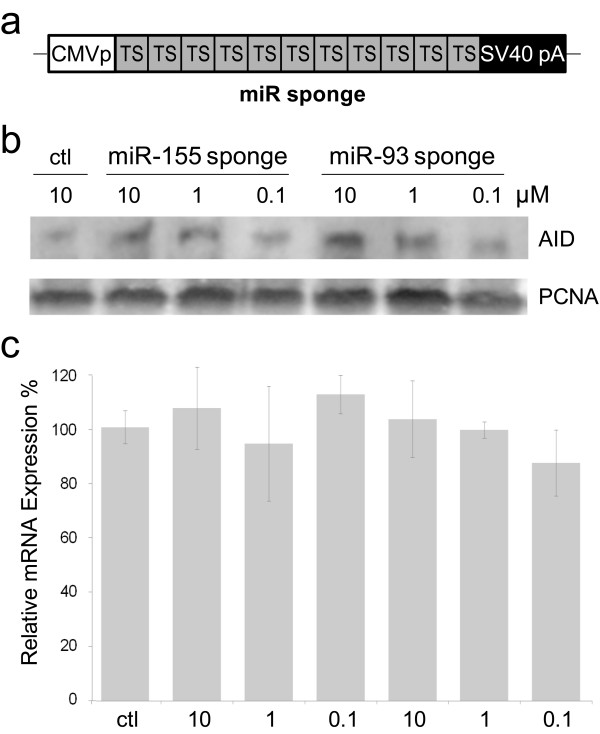
**Endogenous miR-93 and miR-155 restrain AID protein expression**. (**A**) Diagram of miR sponge expression vector. TS indicates an *AID *3'UTR miR target site detailed in **1A**. The miR-93 sponge produces a polyadenylated concatamer of 11 individual 93 TSs. Analogously designed, the miR-155 sponge contains 10 individual miR-155 TSs, and the ctl sponge contains 14 individual shLacZ targets. CMVp, immediate early cytomegalovirus promoter; SV40pA, simian virus 40 polyadenylation signal. (**B**) Representative western demonstrating that transient transfection of MCF-7 cells with the miR-93 or miR-155 sponges results in dose responsive increases in AID protein levels as compared to control. Ctl, shLacZ target site sponge transfected; PCNA, Proliferating Cell Nuclear Antigen. (**C**) Relative *AID *mRNA levels corresponding to replicate transfections performed as in **B **(n = 3) as determined by quantitative PCR.

Over-expression of miR-93 and miR-155 repressed the expression of endogenous *AID*, supporting the model that both miRs contribute to post-transcriptional regulation of *AID*. To be consistent with that hypothesis, the loss of cellular miR-93 or miR-155 should result in higher *AID *expression. Therefore, we depleted miR-93 and miR-155 individually and then monitored changes to AID protein levels by western blotting. To achieve this, we constructed miR sponge transgenes for miR-93 and miR-155 that function by competitively inhibiting miR activity, as previously described [35,36]. Our miR sponge transgenes produce RNAs containing ~10 copies of the miR-93 or miR-155 *AID *3'UTR complementary binding sites (Figure 4A). Individual miR sponges or a negative control sponge transgene were transiently transfected in MCF-7, and relative AID protein levels measured by western. MCF-7 cells expressing either miR-93 or miR-155 sponges showed clear dose-responsive increases in AID protein levels, whereas control transfections did not significantly alter AID protein expression (Figure 4B). To ensure these findings were the consequence of restricted miR activity, we isolated total RNA from each of our transfections and examined *AID *mRNA levels by quantitative PCR. We found *AID *mRNA levels largely unaffected by transfection of sponge constructs further supporting endogenous roles for these miRs in the restraint of *AID *translation (Figure 4C). We conclude that one function of endogenous miR-93 and miR-155 expression is to restrict the translation of *AID *mRNA.

## Discussion

### Model for *AID *repression

**Figure 5:**
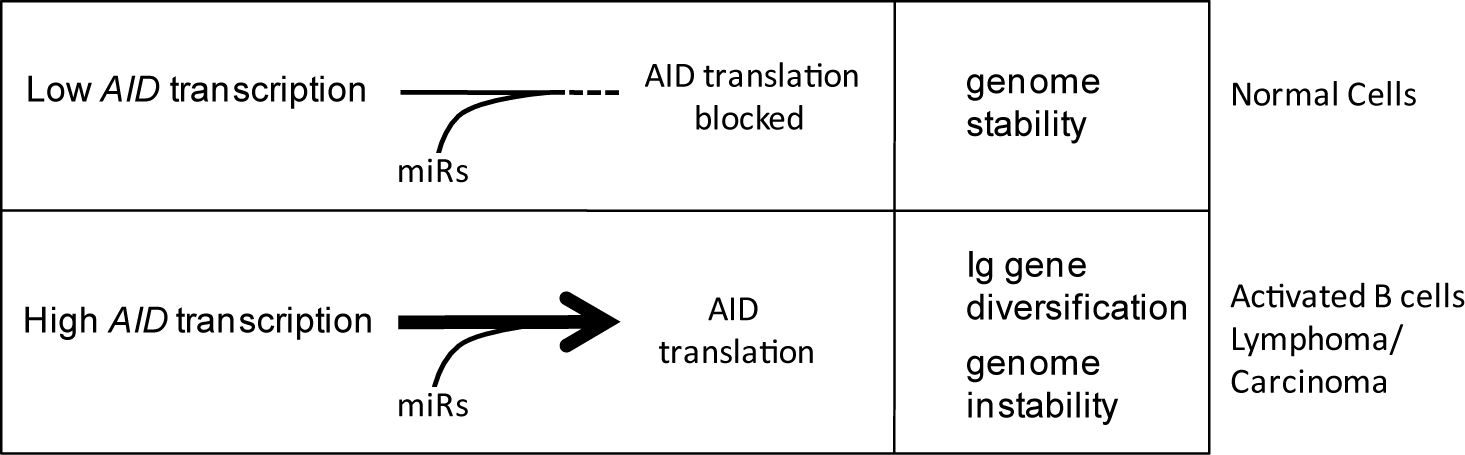
**Model of miR-93 and miR-155 regulation of AID mediated genomic instability**. In normal cells, top, genome stability is maintained by inhibition of AID translation by miR-93 and/or miR-155 when *AID *mRNA levels are low. Bottom, in antigen activated B cells or when misregulated, levels of *AID *mRNA exceed the capacity for miR translational repression. *AID *translation results in AID protein levels that support Ig gene diversification, mutagenesis and genome instability.

We have shown that miR-93 and miR-155 have the capacity to repress *AID *translation and do so in the cellular context. Overexpression of miR-93 or miR-155 reduced AID protein levels in MCF-7 breast cancer cells (Figure 3A), whereas depletion of either endogenous miR-93 or miR-155 resulted in increased *AID *translation (Figure 4) directly connecting miR expression with changes in AID protein concentration. Based on our findings, we propose a model for the relationship between miRs -93 and -155 and *AID *regulation in maintaining genomic integrity (Figure 5). In cellular circumstances where high levels of *AID *transcription exist (such as antigen activated B cells or some established cancers) *AID *mRNA levels overwhelm miR controls. In these situations, endogenous miR-155 and miR-93 have negligible influence on AID protein levels. In contrast, miR-93 and miR-155 can greatly reduce the potential for errant *AID *expression which protects the genome from unwanted cytidine deamination and mutation by restricting AID protein production (Figure 5). This may be particularly important during S-phase when replication transiently denatures the DNA. Conspicuously, miR-93 is intronically encoded within the minichromosome maintenance 7 (*MCM7*) gene, an essential replication licensing factor, and precursor miR-93 abundance tracks with *MCM7 *expression levels [37,38]. It is therefore tempting to speculate that domestication of the miR-93 microRNA along with the corresponding *AID *3'UTR site occurred as a response to the selective pressure for maintaining genome fidelity during replication.

### Disruptions of miR-155 and miR-93 are associated with AID-induced oncogenesis

Highlighting the importance of tightly controlled *AID *expression, novel *AID *regulations continue to be described. In 2008, miR-155 was identified as an important repressor of *AID *translation in mice, and loss of this regulation is lymphomagenic [29,30]. Our data now demonstrate directly that expression of endogenous miRs -93 and -155 represses *AID *translation in MCF-7 breast cancer cells. While a direct correlation between miR-93 activity and AID-induced oncogenesis remains to be described, miR-93 perturbations have been found to enhance cell survival, possess oncogenic activities, and augment tumor growth through regulating integrin-β8, the tumor suppressor gene FUS1, the Cdk inhibitor p21, and tumor protein 53-induced nuclear protein 1 (TP53INP1) [39-42]. Our results suggest that in addition to these known oncogene regulations, loss of miR-93 (or miR-155) may permit *AID *upregulation and mutator phenotypes in non-B cell oncologies (e.g. breast, colon, stomach and lung [21-24]). However, it is possible that additional miR AID regulations may still be described, and our research does not seek to eliminate other potential regulators.

### MicroRNA target sites obscured by repetitive element insertions

Our identification of an extensive and uncharacterized 3'UTR for human *AID *reveals the presence of sequence elements that may confound miR regulatory relationships for other mRNAs. We find that the *AID *3'UTR is ~1000 nt longer than previously annotated likely due to mispriming from an internal stretch of 20 adenosines. The full length *AID *3'UTR contains a previously unidentified regulatory sequence, an unusually pronounced miR-93 complementarity, (Figure 1) conferring marked repression to targeted transcripts (Figure 2 and Additional File 2, Figure S1). Clearly, this finding agrees with recent suggestions that cryptic miR complementarities may actually be quite common with nearly 50% of human and mouse 3'UTRs likely extending well beyond their annotated termini [43,44]. Since 3'UTRs contain miR binding sites, our analysis of *AID *is consistent with the notion that other miR posttranscriptional regulations may have been overlooked because of truncated 3'UTR annotations. This may be particularly pronounced in humans as 5-10% of all human 3'UTRs contain at least one Alu repeat, a common miR-associated retro-element characterized by a central 10-40 nt adenosine linker [32,45-51].

## Conclusions

In the present study, two human microRNAs, miR-155 and miR-93, were each shown to repress the translation of human *AID *through interactions with the *AID *3'UTR in the MCF-7 breast carcinoma cell line which aberrantly expresses *AID*. Together, our data suggest that low-level errant *AID *expression and subsequent genome damage may be prevented through protective miR-93 and/or miR-155 regulation. In addition, our identification of a miR-93 target site for *AID *located downstream of an internal adenosine repeat highlights the possibility that other miR regulations may have been overlooked because of truncated 3'UTR annotations.

## List of abbreviations

AID: Activation Induced Deaminase; bp: base pair; ChIP: chromatin immunoprecipitation; EST: expressed sequence tag; HEK: human embryonic kidney; Ig: immunoglobulin; MCF-7: Michigan Cancer Foundation - 7 breast cancer line; miR: microRNA; mRNA: messenger RNA; nt: nucleotide; pAL: Alu promoter; TS: microRNA target site; UTR: mRNA untranslated region; 3'LR: 3'UTR luciferase reporter.

## Competing interests

The authors declare that they have no competing interests.

## Authors' contributions

GMB: experimental design, experimentation, and manuscript preparation, NWH: experimentation, EDL: research design and manuscript preparation. All authors read and approved the final manuscript.

## Pre-publication history

The pre-publication history for this paper can be accessed here:

http://www.biomedcentral.com/1471-2407/11/347/prepub

## Supplementary Material

Additional file 1 **Table S1. Oligonucleotide master list**. All oligonucleotides were synthesized commercially (Fisher, Operon) at either 25 or 100 nM scale. "pAL" refers to the human miR-517a Alu promoter. "93" and "155" refer to human miRs. "TS" refers to a siRNA or miR target site. ^1^, lowercase indicates imperfect antisense. ^2^, AID 3'LR was generated using TS-155F and TS-93R. ^3^, designed previously [See reference [32]]. Click here for file

Additional file 2 **Figure S1. The *AID *3'UTR is recognized by miRs -93 and -155**. (**A**) The AID 3'UTR recognition by miRs -93 and -155 is specific. (**A**) Diagrams of luciferase reporters. The AID 3'UTR luciferase reporter (TS Intact) and control 3'UTR luciferase reporter (TS Scram) were generated respectively by placing the AID 3'UTR miR -93 and -155 target sites (TS Intact)(with intervening sequences deleted) or an identical sequence in which the target site nucleotides had been scrambled (TS Scram) into a multiple cloning site in the Renilla luciferase 3'UTR [32]. HSV-TKp, herpes simplex virus thymidine kinase promoter; SV40p, simian virus 40 promoter; FF Luc, firefly luciferase; Ren Luc, Renilla luciferase; pA, poly(A); 155TS, miR-155 target site; 93TS, miR-93 target site; 155SC, miR-155 target site scrambled; 93SC, miR-93 target site scrambled. (**B**) TS Intact is specifically repressed by both miR-93 and miR-155 in 293 transient transfections. Luciferase assays (n = 9) of HEK293 lysates after transfection of TS Intact and either pAL-1, pAL-155 or pAL-93. Transfections were normalized to TS Intact alone. RLU, relative light units. (**C**) TS Scram is unaffected by co-transfection of pAL-1, pAL-155 or pAL-93 in 293 transient transfections. Cotransfections done as in **B**, except TS Scram replaced TS Intact. Click here for file

Additional file 3 **Figure S2. MicroRNAs miR-93 and miR-155 are widely expressed**. RT-PCR of pre-miR template in three different cell lines, HEK293 embryonic kidney cells, MCF-7 breast cancer line, and Ramos Burkitt's lymphoma line. (**top**) RT-PCR products of the miR-93 locus amplified from indicated cellular RNAs. m, miR-93; β, Beta actin positive control; - Ctl, no template. (**bottom**) RT-PCR products of the miR-155 locus amplified from indicated cellular RNAs. Annotations are same as above except (m) denotes pre-miR-155 amplicon. PCR of source RNAs failed, indicating above RT-PCRs templated from the generated cDNA. Click here for file

Additional file 4 **Figure S3. MiR-93 represses endogenous AID in a dose responsive manner**. (**A**) Representative western demonstrating that transient transfection of pAL-93 expression constructs repress endogenous AID protein levels in MCF-7 cells in a dose responsive manner. (**B**) Relative AID mRNA levels corresponding to replicate transfections performed as in **A **(n = 2) determined by quantitative PCR. Click here for file
