# Supplementary material for: Repression of human activation induced cytidine deaminase by miR-93 and miR-155
Source: BMC Cancer. 2011 Aug 10;11:347. doi: 10.1186/1471-2407-11-347 (PMC3163633; doi:10.1186/1471-2407-11-347)
Supplement: Additional file 1 — Table S1. Oligonucleotide master list. All oligonucleotides were synthesized commercially (Fisher, Operon) at either 25 or 100 nM scale. "pAL" refers to the human miR-517a Alu promoter. "93" and "155" refer to human miRs. "TS" refers to a siRNA or miR target site. 1, lowercase indicates imperfect antisense. 2, AID 3'LR was generated using TS-155F and TS-93R. 3, designed previously [See reference [32]]. [file 1471-2407-11-347-S1.PDF]

**pAL Construct Primers**	
5’Alu Forward[3]	CCCAAGGACACCCTCATGATCTCCC
3’pAL-93R	AAAAACTCGGGAAGTGCTAGCTCAGCAGTAGGTTGGGTAATCACACTACCTGCACGAACAGCACTTTGGAGTGGATATCTGCAGAATTCG
3’pAL-155R	AAAAAACTGTTAATGCTAATATGTAGGAGTCAGTTGGAGGCAAAAACCCCTATCACGATTAGCATTAACAGGGATATCTGCAGAATTCG
3’pAL-1R[3]	AAAAACGTGACCTATCCCATTACGTGACAGGAAGCGTAATGGGATAGGTCACGTTGATGGATATCTGCAGAATTCG
3’pAL-shAIDr	AAAAAAAAATGTCCGCTGGGCTAAGGTGACAGGAAGCCTTAGCCCAGCGGACATTTGATGGATATCTGCAGAATTCG
**Sponge Construct Primers**	
**93 Sponge**	
Gb130SPG93TSf	GGTGAATATAACGCCTGTAATCCCAGCACTTTGGGAGGTGAATATA
Gb131SPG93TSr	TATATTCACCTCCCAAAGTGCTGGGATTACAGGCGTTATATTCACC
**155 Sponge**	
Gb132Spg155TSf	GGTGAATATATGGGGATAGTGCTAATCGTAATTGGTGAATATA
Gb133Spg155TSr	TATATTCACCAATTACGATTAGCACTATCCCCATATATTCACC
**Sponge Control**[1]	
Gb140spgsiLacZ5p10f	GGTGAATATATTaaTACGTGACCTATTaaTGGTGAATATA
Gb141spgsiLacZ5p10r	TATATTCACCAttAATAGGTCACGTAttAATATATTCACC
**AID 3’UTR Target Sites and Reporter Primers**	
**MiR-93-TS**	
TS-93F	GGAAGGAAGTTGCTTGAATGTTGG
TS-93R[2]	GAGACAGAGTCTTGCTCTTGTCG
**MiR-155-TS**	
TS-155F[2]	CCTGGGAGCATCCTAAAGTGTCAAC
TS-155R	TCTTGTTCACAGTCCTCCTGCCC
**Ctl 3’LR**[3]	
LacZ_TS_F	TTCTCGAGCGGTTACGATGCGCCCATCTACACCAACGTGACCTATCCCATTACGGTCAA
LacZ_TS_R	AAGCGGCCGCGGGAACAAACGGCGGATTGACCGTAATGGGATAGGTCACGTTGGTGT
**TS Intact**	
TSIntactF	AAACTCGAGTGATTGTGACCCCAAACCATCTCTCCAAAGCATTAATATCCAATCATGCGCGGCGTGGTGGCTC
TSIntactR	TTTGCGGCCGCGCTCGGCCTCCCAAAGTGCTGGGATTACAGGCGTGAGCCACCACGCCGCGCATGATTGG
**TS Scram**	
TSScramF	AAACTCGAGTGATTGTGACCCCATCTATCAATCAACCTAAGAACTCATCCAATCATGCGCGGCGTGGTGGCTC
TSScramR	TTTGCGGCCGCGCTCGGCCTCGGTTGAACGGTAACGGCAGACTCTGAGCCACCACGCCGCGCATGATTGG
**RT-PCR Primers for miR Expression**	
**Pre-miR-93**	
miR-93F	GAGATGAGGCGAGAGGCTTGGG
miR-93R	CCTTTGACATCTCCATTAGCCTGATGG
**Pre-miR-155**	
miR-155F	GCCTCCAACTGACTCCTACATATTAGC
miR-155R	GTTTAAGGTTGAACATCCCAGTGACCAG
**β Actin**	
gb204_bAct2F2_62	CCAACCGCGAGAAGATGACCC
gb205_bAct2R1_61	CTCCTTAATGTCACGCACGATTTCCC
**Quantitative PCR Primers**	
**AID**	
AID-F	GTGACCCCAAACCATCTCTCCAAAGC
AID-R	TCCCCAACATTCAAGCAACTTCCTTCC
**β Actin**	
bActF	GGCTCCGGCATGTGCAAGG
bActR	GGTGAGGATGCCTCTCTTGCTCTG
**tRNAglu**	
trna_glu_F	AAGGCGCATCTCTAGTTCAGTGG
trna_glu_R2	CTCTTTGCTCCTGGAGTCTCTCAC

Additional File 1, Table S1. Oligonucleotide master list. All oligonucleotides were synthesized commercially (Fisher, Operon) at either 25 or 100 nM scale. “pAL” refers to the human miR-517a Alu promoter. “93” and “155” refer to human miRs. “TS” refers to a siRNA or miR target site. [1], lowercase indicates imperfect antisense. [2], AID 3’LR was generated using TS-155F and TS-93R. [3], designed previously [See reference 32]
